# The effect of health literacy on health status among residents in Qingdao, China: a path analysis

**DOI:** 10.1186/s12199-021-01001-8

**Published:** 2021-08-12

**Authors:** Yiqing Huang, Fei Qi, Rui Wang, Xiaorong Jia, Yani Wang, Peng Lin, Meiyun Geng, Shanpeng Li

**Affiliations:** 1 Department of Epidemiology and Health Statistics, The School of Public Health of Qingdao University, Qingdao, China; 2 Qingdao Municipal Center for Disease Control and Prevention, Qingdao, Shandong China

**Keywords:** Health literacy, Self-efficacy, Health behavior, Health status, Path analysis

## Abstract

**Background:**

Health literacy is a public health goal which can be used as an independent factor of health outcomes. This study aimed to assess the association between health literacy and health status, as well as the two mediating factors of behavior and self-efficacy among residents aged 15–69 years in Qingdao.

**Methods:**

A cross-sectional survey was implemented among residents aged 15–69 years (*N* = 3793) in Qingdao, China. A combination of stratified cluster random and proportional probability sampling methods was used to select subjects for this study. Data were collected using “The Chinese Citizen Health Literacy Questionnaire (2019)”. We proposed a hypothetical model for the relationship between sociodemographic characteristics, health literacy, self-efficacy, health behavior, and health status, and used path analysis to validate the hypothesis.

**Results:**

The path analysis showed that higher education (*β* = 0.293) and income (*β* = 0.135) are positively and directly associated with greater health literacy, which was positively associated with health status (*β* = 0.057). Health literacy is a direct influencing factor of health behavior (*β* = 0.070) and self-efficacy (*β* = 0.099). Health behavior (*β* = 0.041) and self-efficacy (*β* = 0.173) exerted a positive direct effect on health status. The model explained 14.1% of variance for health literacy, 3.8% for self-efficacy, 5.7% for health behavior, and 15.0% for health status.

**Conclusions:**

Health literacy was identified to be a critical factor in health status. The results emphasized that the dissemination of health knowledge, development of healthy behavior, and cultivation of self-efficacy should be jointly promoted to reinforce the level of health status among residents in future work.

## Introduction

Health literacy is defined as the capacity of individuals to access, process, and understand basic health information and services, and use them to make proper health decisions [1]. Approximately 39% of people worldwide lack health literacy, whereas only 17% of the population in China show adequate health literacy [2, 3]. Health literacy is considered a major public health goal that can be used as an independent influencing factor of health outcomes [4]. In addition, people with insufficient health literacy may incur high medical expenditures and cause substantial financial burden [5]. Therefore, health literacy has become an important indicator for the measurement of health status among residents. Improving health literacy is a fundamental, economical, and practical measure to enhance the health of people in general.

Previous evidence has shown that a causal relationship exists between health literacy and health status among different populations. Generally speaking, health literacy is a key factor for patients with chronic diseases in controlling their condition and achieving positive health outcomes [6]. People with deficient health literacy may experience 1.5–3 times more serious health outcomes such as higher mortality [7], hospitalization rates [8], and inferior disease management ability [9] than those with sufficient health literacy [7, 10]. Health literacy can also indirectly impact health status by affecting health behavior [11]. A British study revealed that limited health literacy was associated with negative behavior in life [12]. People with sufficient health literacy are less likely to smoke, exercise more frequently, and self-rate their health status better than those with deficient health literacy [12]. Furthermore, the effect of health literacy on health status can be explained by the role of psychosocial factors, such as self-efficacy [13]. Self-efficacy refers to the subjective judgement of individuals on whether they can successfully perform a specific behavior [14]. Although some studies have explored the association among health literacy, self-efficacy, behavior, and health status, the results are inconsistent. Some studies have demonstrated that the lack of self-efficacy [13] or poor health behavior [15, 16] of people with insufficient health literacy can lead to adverse health outcomes [17], while other studies have reported null associations [18–20]. Some investigations have also discovered that self-efficacy is associated with health behavior or life quality, while other studies did not find such associations [20–22]. Differences in these intermediate factors may lead to different roles played by health literacy in improving health outcomes.

Baker proposed a conceptual model that showed how health literacy affects health status [23]. This model assumes a cascading causal process in which health literacy will affect health changes through multiple paths. Health literacy affects health status through health behavior and/or psychological pathways. Health behavior and self-efficacy were considered important mediators between health literacy and health status. In addition, Baker also believed that the model should consider the impact of sociodemographic variables on health literacy and health status [23]. Besides, Paasche-Orlow also established a conceptual framework for individual health literacy [24]. The framework illustrated that sociodemographic characteristics were basic factors for health literacy and outlined pathways in which health literacy may influence health outcomes. Therefore, the above relationship is the basis of the theoretical framework of the present study.

Healthy China Action clearly stated that the improvement of health literacy is a prerequisite for improving the health of all people [25]. However, existing studies in the literature related to health literacy among Chinese residents have not been well appreciated. The internal mechanism of how health literacy affects the health status of Chinese residents remains unclear. Therefore, building on Baker’s and Paasche-Orlow’s models, we proposed a model that contained five key elements, including, sociodemographic variables, health literacy, self-efficacy, health behavior, and health status. The purpose of this study is to analyze the path relationship between health literacy and health status among residents aged 15–69 years in Qingdao and consider the contribution of health behavior and self-efficacy in the pathway. We hypothesized that adequate health literacy not only exerted a positive influence on health status directly but also indirectly contributed to health status through self-efficacy or health behavior. Meanwhile, self-efficacy exerts a positive effect on health and can also affect health status through health behavior.

## Methods

### Study design and sampling

The target population was residents aged 15–69 years who have lived in Qingdao for more than 6 months within a year, excluding residents living in military bases, hospitals, prisons, nursing homes and dormitories, and other places, accounts for approximately 7 million of the population. The combination of multi-stage stratified cluster and proportional probability sampling (PPS) of the permanent population was conducted in our study. A certain number of neighborhood committees have been selected as monitoring sites according to the number of households included in each neighborhood committee after stratified sampling of urban and rural areas. Forty communities comprising 24 urban and 16 rural neighborhoods were selected. The number of households in each neighborhood is between 750 and 1500. Each monitoring site obtains a complete household sampling frame through mapping and listing, and 100 households are randomly sampled from each sampling frame. The investigator used the KISH table to select a random member aged 15–69 years from each household and conducted the survey.

The sample size was calculated by using the function $N={{\mu }_{\alpha }}^{2}P(1-P)/{\delta }^{2}\times deff$. According to the data obtained from the Qingdao Health Literacy Survey in 2017, the expected percentage was 15.92% (*P* = 0.1592) [26]. Given that the invalid questionnaire and rejection rate do not exceed 20%, the minimum sample size should be at least 1929. A total of 4000 people were eventually recruited on the basis of the proportion of urban and rural populations in Qingdao. The purpose of accomplishing the questionnaire was explained, and an informed consent form was signed by participants. Participants under 16 years are signed by their parents. Residents filled out the questionnaire independently using an application installed on the pad.

## Measures

### Sociodemographic characteristics

We incorporated three sociodemographic characteristics of age, education level, and annual household income into the path analysis as control variables by reviewing the previous literature. Participants reported their highest education level (1 = elementary school and below, 2 = junior high school, 3 = high school/vocational high school/technical secondary school, and 4 = bachelor degree and above), annual household income (1 =  < 10,000 CNY, 2 = 10,000–30,000 CNY, 3 = 30,000–50,000 CNY, 4 = 50,000–100,000 CNY, 5 =  > 100,000 CNY), and age in the questionnaire.

### Health literacy

Health literacy was assessed using the Chinese Citizen Health Literacy Questionnaire (2019), which was developed by the China Health Education Center. It was compiled by experts from multiple disciplines based on the “Basic Knowledge and Skills of National Health Literacy (2015)” using the Delphi method [27]. A comprehensive index or percentage was applied to the questionnaire to evaluate health literacy and reflect the health knowledge and skill among residents accurately. In this study, comprehensive scores were used to assess health literacy. The 56-item questionnaire included true-or-false, single-choice, and multiple-choice questions, with a total score of 73 points. Every correct answer gets 1 point for true-or-false and single questions, while that for multiple-choice questions gets 2 points, and every wrong or missing choice gets 0 point. Adequate health literacy is achieved when participants reach more than 80% of the total score. High scores indicated that residents present adequate health literacy and abundant health knowledge. Moreover, the questionnaire showed good internal consistency (Cronbach’s *α* = 0.903).

### Self-efficacy

The self-efficacy scale is based on the Chinese version of the general self-efficacy scale [28]. Core questions can be selected to represent the self-efficacy of participants due to the limited length of the questionnaire. The self-efficacy scale includes three questions, including “I can always solve problems”, “I can stick to my ideals and achieve goals”, and “I can make my own decisions in general”. All three self-efficacy items are rated on using a five-point Likert scale ranging from 1 (strongly disagree) to 5 (strongly agree) to measure whether an individual can successfully perform a subjective judgement of behavior. Scores for each option are added to calculate the total number of points, ranging from 3 to 15. The higher score indicates that people are capable of or confident in completing health-related behaviors.

### Health behavior

Health behavior was evaluated according to smoking, drinking, physical exercise, sleep duration, and physical examination. Participants chose one of the options from each question to present their actual situation. Original options were reclassified, and values were assigned in accordance with common recommendations in the previous literature on smoking, drinking, physical exercise, sleep duration, and physical examination to facilitate the analysis. The smoking categories were ‘current smoking’, ‘quit smoking’, and ‘never smoking’. The drinking categories were ‘daily’, ‘sometimes’, and ‘never’. The physical exercise categories were ‘yes’ (do exercise in the past week) versus ‘no’ (not do exercise in the past week). The sleep duration categories were ‘7–8 h’ versus ‘non 7–8 h’. The physical examination categories were ‘within a year’, ‘1–2 years’, ‘3 years and above’, and ‘never’. The total score (a minimum of 0 points to a maximum of 9 points) was used as the behavior score. A high behavior score reflects the positive health behavior of participants.

### Health status

Health status was measured using the EuroQol-visual analogue scale (EQ-VAS) [29]. Participants provided a self-rating on the vertical scale, with 100 points for “best health conditions” and 0 points for “worst health conditions”. On the basis of their actual situation, a high self-rating score indicates an improved health condition. Self-rated health status is a simple and important evaluation index derived from a series of screening, evaluating, and summarizing of information process from individuals and consciousness [30]. This index can not only reflect the personal health status but also integrate its subjective and objective aspects [31]. Although the reliability of health self-evaluation is still controversial in reflecting health status, the majority of studies have shown that self-rated health can be used as a robust and reliable indicator of individual health status [32].

### Statistical analysis

A descriptive analysis of general characteristics of residents in Qingdao was performed using SPSS 24.0. Given that the variance inflation factor (VIF) of each variable was less than 5 and the bivariate correlation did not exceed 0.80, there was no multicollinearity between the variables and no violation on the assumption of mutual independence. In addition, the skewness coefficient was less than 3, and the kurtosis coefficient was less than 8, so the data met the assumption of normal distribution. On the basis of previous studies, we regarded age, education level, and annual household income as exogenous variables, while health literacy, self-efficacy, health behavior, and health status were used as endogenous variables for constructing the original structural equation model. Path analysis was performed to test the relationship between sociodemographic characteristic, health literacy, health behavior, self-efficacy, and health status among residents aged 15–69 years in Qingdao. The model parameters were estimated using the maximum likelihood method. Standardized regression coefficients were utilized to evaluate the influences of sociodemographic variables, health literacy, self-efficacy, and health behaviors (smoking, drinking, physical exercise, sleep time, and physical examination) on health status in the path model. The overall fitness of the model was evaluated using the comparative fit index (CFI), Tacker-Lewis index (TLI), and root mean square error of approximation (RMSEA). The model was considered acceptable because both CFI and TLI values were greater than 0.95, and RMSEA was less than 0.05 [33]. We trimmed insignificant paths to adjust the path model until the main goodness-of-fit index indicated that the final model fitted the data appropriately. The effects of the path analysis were decomposed, and the direct and indirect effects between variables were calculated. Path analysis, including the estimation of path coefficients and the assessment of the overall fit of the structural model, was carried out using Mplus 8.3. All *P* values were two-tailed, and the level of significance was set at a *P* value less than 0.05..

## Results

A total of 3793 residents filled out the questionnaire, with an effective response rate of 94.8% (3793/4000). Half of the 3793 respondents were male (50.3%). The age of participants ranged from 15 to 69 years, and the average age was 49.69 ± 13.2 years. The marriage status demonstrated that the majority of participants were married (83.6%), with the remaining proportions of the single and widowed participants were 11.8%, and 4.6%, respectively. Occupations of the residents involved many fields, such as worker (19.3%), office worker (including civil servant, teachers, doctors, and institution officers) (32.6%), farmer (38.9%), student (2.0%), and others (7.3%). Over 50% of the residents reported their annual household income more than 30,000 CNY. The mean scores for health literacy, self-efficacy, health behavior, and self-rated health status were 45.80 ± 13.81, 13.73 ± 1.31, 6.03 ± 2.04, and 85.38 ± 11.78, respectively. The descriptive characteristics of the population are shown in Table 1.

**Table 1:** Descriptive statistics of the participants (*N* = 3793)

	***N* (%) or mean (SD)**
Age (years)	49.69 (13.22)
Gender	
Male	1909 (50.3)
Female	1884 (49.7)
Education	
Primary school and below	668 (17.6)
Junior high school	1371 (36.1)
High/professional high/technical secondary school	850 (22.4)
College/bachelor and above	904 (23.8)
Marital status	
Single	448 (11.8)
Married	3172 (83.6)
Widowed	173 (4.6)
Occupation	
Office worker	1237 (32.6)
Student	74 (2.0)
Farmer	1476 (38.9)
Worker	731 (19.3)
Others	275 (7.3)
Annual household income (CNY)	
< 10,000	287 (7.6)
10,000–30,000	694 (18.3)
30,000–50,000	656 (17.3)
50,000–100,000	1295 (34.1)
> 100,000	861 (22.7)
Health literacy (score)	45.80 (13.81)
Self-efficacy (score)	13.73 (1.31)
Health behavior (score)	6.03 (2.04)
Health status (score)	85.38 (11.78)

The bivariate correlation matrix displayed that health literacy and health status are significantly associated with all variables included in the model (Table 2). Health literacy was significantly correlated with all other variables, but the strongest correlation was with education (*r* = 0.356). In addition, correlation among self-efficacy and health status was strong (*r* = 0.232), while health behavior presented a slightly strong correlation with self-efficacy (*r* = 0.084) and age (*r* =  − 0.084).

**Table 2:** Correlation matrix of the variables in the model

	(1)	(2)	(3)	(4)	(5)	(6)	(7)
(1) Age	1.000						
(2) Education	− 0.518^**^	1.000					
(3) Income	− 0.301^**^	0.468^**^	1.000				
(4) Health literacy	− 0.212^**^	0.356^**^	0.272^**^	1.000			
(5) Self-efficacy	− 0.132^**^	0.140^**^	0.142^**^	0.141^**^	1.000		
(6) Health behavior	− 0.084^**^	0.204^**^	0.189^**^	0.145^**^	0.084^**^	1.000	
(7) Health status	− 0.282^**^	0.247^**^	0.263^**^	0.172^**^	0.232^**^	0.110^**^	1.000

^**^P < 0.01

**Fig. 1:**
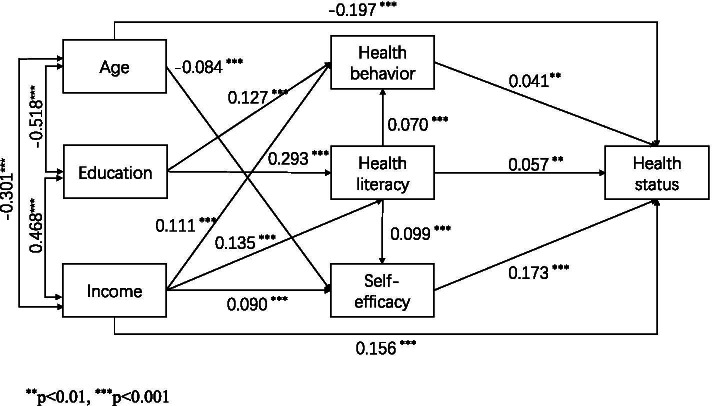
Path analysis with standardized coefficient in the final model

On the basis of these results, we developed a structural equation model to examine the connection between sociodemographic indicators, health literacy, health behavior, self-efficacy, and health status. The final model reached the optimal fit with the existing data after we removed insignificant paths and those that violate the hypothesis. Model fitness indexes revealed an adequate fit, with a model fit of *χ*^2^ = 20.862 (df = 5, *P* = 0.0009), CFI = 0.990, TLI = 0.964, RMSEA = 0.029, and SRMR = 0.013. The estimates of each path are listed in Table 3. As conveyed in Table 4 and Fig. 1, in addition to the direct association, it also has an indirect effect on health status through health behavior or self-efficacy. However, we did not find the relationship between self-efficacy and health behavior. Besides, direct or indirect associations were also observed between sociodemographic variables and health status. A positive health condition was more likely to achieve in residents with higher annual household income than those with lower annual household income when only the direct path was considered. Conversely, older residents are less likely to demonstrate an improved health status. The final model suggested that the impact of education on health status was completely mediated by health literacy, self-efficacy, or health behavior, but a direct connection is absent by themselves. Health literacy, self-efficacy, health behavior, and health status explained 14.1%, 3.8%, 5.7%, and 15.0% of the total variance, respectively.

**Table 3:** Path coefficients based on the final model

**Path**			**Unstandardized coefficient estimate**	**S.E**	***P* value**	**Standardized coefficient estimate**
Health literacy	←	Education	3.893	0.226	< 0.001	0.293
Health literacy	←	Income	1.508	0.195	< 0.001	0.135
Self-efficacy	←	Age	− 0.008	0.002	< 0.001	− 0.084
Self-efficacy	←	Income	0.096	0.019	< 0.001	0.090
Self-efficacy	←	Health literacy	0.009	0.002	< 0.001	0.099
Health behavior	←	Education	0.250	0.037	< 0.001	0.127
Health behavior	←	Income	0.182	0.031	< 0.001	0.111
Health behavior	←	Health literacy	0.010	0.003	< 0.001	0.070
Health status	←	Age	− 0.175	0.015	< 0.001	− 0.197
Health status	←	Income	1.491	0.179	< 0.001	0.156
Health status	←	Health literacy	0.049	0.015	0.001	0.057
Health status	←	Self-efficacy	1.557	0.147	< 0.001	0.173
Health status	←	Health behavior	0.237	0.089	0.007	0.041

**Table 4:** Indirect pathways in the final model

**Pathways**	**Standardized coefficient**	***P* value**
Age → Self-efficacy → Health status	− 0.014	< 0.001
Education → Health literacy → Health status	0.017	0.002
Education → Health behavior → Health status	0.005	0.010
Education → Health literacy → Health behavior → Health status	0.001	0.034
Education → Health literacy → Self-efficacy → Health status	0.005	< 0.001
Income → Health literacy → Health status	0.008	0.003
Income → Health behavior → Health status	0.005	0.013
Income → Self-efficacy → Health status	0.016	< 0.001
Income → Health literacy → Health behavior → Health status	< 0.001	0.038
Income → Health literacy → Self-efficacy → Health status	0.002	< 0.001

## Discussion

The present study showed that sociodemographic indicators, health literacy, self-efficacy, and health behavior were identified as important factors of health status. Path analysis displayed that the association between health literacy and health status occurs through three different pathways. The first pathway directly connected health literacy and self-rated health status, another went through self-efficacy, and the remaining paths were through health behavior. In addition, age and income exerted a direct influence on health literacy. Sociodemographic variables explained 14.1% of variability in health literacy, while health literacy and other variables explained 15.0% of the variability in health status.

This tool we used to assess health literacy is designed to measure an individual’s basic knowledge and concepts, healthy lifestyles and behaviors, and basic skills. Different from foreign health literacy evaluation systems, this public health-oriented tool mainly evaluates people’s ability to obtain, understand, and use health information and is unsuitable for rapid assessment of patients’ health literacy in the medical environment. The proposed tool also follows the World Health Organization’s definition of health literacy, that is, health literacy represents cognitive and social skills that determine the individual’s motivation and ability to obtain, understand, and use health information while promoting health through these pathways [34].

Health literacy in the model was influenced by the two sociodemographic variables of income and education. Similar to the results of Suka [35], health literacy increased with income. Notably, high income and health status were partially related to adequate health literacy, strong self-efficacy, and positive behavior. This finding further verified that this relationship may also be partially due the susceptibility of residents to health literacy, self-efficacy, and behavior in addition to the direct impact of high income on health status. These findings emphasized the importance of considering complex models that include different aspects of residents’ lives to understand the ways in which positive health conditions are formed further. Consequently, interventions aimed at developing positive health conditions should not be limited to measures for increasing family income because income will only slightly fluctuate over a long period of time. Meanwhile, other approaches should be considered to address residents with poor health literacy and the resultant low self-efficacy or risk behavior.

In terms of the relationship between education and health literacy, our results were also similar to Sun’s [11]. Education was related to self-rated health status through health literacy and its consequence. On the basis of this premise, the residents will unlikely demonstrate sufficient health literacy when they present a low education level. This finding may spiral into low self-efficacy and negative health behavior, and eventually lead to serious health outcomes when ignored. The current results also support the evidence from a previous study, which reported that health literacy may be a pathway for socioeconomic status to affect health status, especially in low socioeconomic groups [36]. Therefore, health literacy may be easier to modify than the main established socioeconomic determinants of health inequality. These findings reinforce the claim that the lack of health literacy among vulnerable groups plays a fundamental role in individuals’ health, thereby indicating that the problem of poor health status must be addressed through multi-level interventions implemented by different professions and departments.

Our results demonstrated that a positive effect existed between health literacy and self-rated health status. The significance of health literacy in the process was noticeable. This finding has repeatedly recurred in the literature over the years [37, 38]. Baker proposed that health literacy was one of the many factors that could contribute to improved health outcomes [23]. Besides, broadly defined health literacy also included conceptual health knowledge [23]. People with sufficient health literacy showed the initiative and enthusiasm to acquire their own health information and core knowledge of diseases while adopting corresponding health skills in daily life to seek help from others according to their own characteristics. Meanwhile, they can use social resources to enhance healthy behaviors and reduce risk factors for disease, thereby promoting a positive health status. The improvement of basic knowledge, basic skills, and lifestyle literacies will inevitably exert an impact on residents’ cognition, psychology, and health-related behavior. Moreover, positive health conditions are inseparable from the expansion of health knowledge, the formation of healthy lifestyle, or the development of health-related skills [39, 40]. People with adequate health literacy attach great importance to their own health status and autonomously learn relevant health knowledge through the Internet or other pathways to improve their health status. Although several people have already suffered from chronic diseases, the quality of life and disease management competence with sufficient health literacy is better than those with insufficient health literacy due to the mastering of knowledge, methods, or skills in dealing with diseases [41, 42].

These findings suggested that health behavior preceded self-rated health status. Consistent with the original hypothesis proposed in the model, health behavior played a mediating role between health literacy and health status. People with sufficient health literacy are more likely to seek health information through multiple channels, which changes people’s perceptions on health issues and influences them to alter their behavior, thereby making decisions that are beneficial to their health [43]. Similarly, individuals with inferior health literacy are less likely to adopt positive behavior and may avoid obtaining health information, thereby increasing health barriers. From this perspective, the behavior is a significant factor in the process between health literacy and health status, thereby indicating that insufficient health literacy is only one of the causes of poor health status.

We also found that health literacy had an active influence on health status through self-efficacy, and this finding was consistent with that of a previous study [44]. Our study indicated that increasing health-related knowledge and addressing related psychosocial factors were necessary to enhance the health status of residents with insufficient health literacy. Increased self-efficacy may promote beneficial results through specific behaviors, such as weight reduction [45], smoking cessation [46], and adherence to exercise programs [47], allowing people to avoid conditions that contribute to serious health outcomes or maintain satisfactory health conditions. However, we did not find the significant connection between self-efficacy and behavior in this study. This finding is inconsistent with our hypothesis. Some researchers postulated that self-efficacy is a predictor factor of behavior [18]. The social cognitive theory proposed that high self-efficacy may be necessary to promote positive behavior [48]. Self-efficacy is an important determinant in deciding to start a new behavior pattern, and the increase of self-efficacy is an essential precursor of behavior change [49–51]. Further research can explore the connection between self-efficacy and health behavior among residents in Qingdao.

This study has several advantages. We used the questionnaire of “2019 health literacy survey of Chinese citizens” for the first time to demonstrate the indirect pathways of health literacy to health status through self-efficacy and health behavior. Furthermore, our statistical method that uses path analysis is superior to linear regression analysis because it explains the relationship between various factors and investigates the direct and indirect relationships among the variables. Although our findings are compatible with those of previous studies, we extended known associations between health literacy and health outcomes with the 2019 Chinese Citizens Health Literacy Questionnaire through path analysis. And the significance of the model we construct was to explain the determinants of health literacy and the relationships between health literacy, self-efficacy, and health behavior. Therefore, the entry point of intervention strategies and important gaps in the pathways linking health literacy and health status can be identified.

However, several limitations of the study should be noted. First, although our findings indicated a causal relationship between variables, the nature of the cross-sectional study fails to draw conclusions about causality. Therefore, we relied on theories and existing literature to guide our findings and explain the relationship between variables over a period of time. Longitudinal influences of these factors on health status are subject to further prospective studies. Second, although the relationships between the variables in our study are statistically significant, the magnitude of the relationships is quite limited. Health literacy and other intermediate variables in the model explain only 15% of the health status of the population. This finding indicated that the differences in self-rated health status may be due to the insufficient measurement of variables or other unmeasured factors influencing health status. Hence, the relationship between health literacy and health status must be fully examined in the future investigation. Finally, our research, especially for health status, relies on self-reported measurement. If residents with high health literacy report high scores as evidence of enhanced health conditions, then effect of health literacy on health status may be overestimated.

The improvement of health literacy can effectively enhance the health status among individuals. Carrying out actions that promote national health literacy to improve health literacy among residents fundamentally is still the primary task of public health construction. In this study, we focused on the health literacy model at an individual level. Further investigation should extend the scope of health literacy beyond the individual and promote changes in the behavior of the whole people. At the same time, exploring the mechanism of health literacy affecting health status and strengthening empirical investigations on the relationship among health literacy, beliefs, behavior, and health outcomes is necessary to provide a reference for enhancing health literacy and promoting the health level among residents. And we would like to develop intervention measures for addressing health literacy and health issues at the target communities rather than at the individual level.

## Conclusions

Health literacy was significantly correlated with health behavior, self-efficacy, and health status. The path analysis proved that people with adequate health literacy may likely show positive living habits and strong self-efficacy and thus report an improved self-rated health status. According to our results, we suggested that health education should focus on the enhancement of health literacy knowledge in order to improve self-efficacy and promote health-related behavior, and thus achieve desired health outcomes. Health educators and health care providers in public health sectors should jointly promote the dissemination of health knowledge, the development of healthy behavior, and the cultivation of health beliefs to reinforce the level of health status among residents.

## Data Availability

The data used and/or analyzed in the current study are not publicly available because restrictions apply to the availability of these data. Government departments allow researchers to use the data for scientific research, but do not allow anyone to share original data publicly. If other researchers need the data used in this study, please apply to Qingdao Municipal Center for Disease Control and Prevention. Data are available from the corresponding author on reasonable requests and with permission of Qingdao Municipal Center for Disease Control and Prevention.

## Abbreviations

PPS: Proportional probability sampling
EQ-VAS: EuroQol-visual analogue scale
VIF: Variance inflation factor
CFI: Comparative fit index
TLI: Tacker-Lewis index
RMSEA: Root mean square error of approximation
